# Correlates of condomless anal sex among men who have sex with men (MSM) in Tijuana, Mexico: The role of public sex venues

**DOI:** 10.1371/journal.pone.0186814

**Published:** 2017-10-24

**Authors:** Shirley J. Semple, Eileen V. Pitpitan, David Goodman-Meza, Steffanie A. Strathdee, Claudia V. Chavarin, Gudelia Rangel, Karla Torres, Thomas L. Patterson

**Affiliations:** 1 Department of Psychiatry, University of California San Diego, La Jolla, California, United States of America; 2 Division of Global Public Health, Department of Medicine, University of California San Diego, La Jolla, California, United States of America; 3 Division of Infectious Diseases, University of California Los Angeles, Los Angeles, California, United States of America; 4 US-Mexico Border Health Commission, Tijuana, Mexico; 5 Agencia Familiar Binacional, A.C., Tijuana, Mexico; Thai Red Cross AIDS Research Center, THAILAND

## Abstract

Condomless anal sex between male partners is the primary risk factor for HIV transmission among men who have sex with men (MSM). Correlates of condomless anal sex have been well-studied in developed countries, but they have received less attention in lower-to-middle income countries (LMIC), where MSM are often subject to stigma, discrimination, intolerance, and even the criminalization of same sex behavior. In Mexico, a LMIC where traditional views on homosexuality are common, HIV prevalence among MSM is high (16.9%), yet little research has been conducted on the correlates of condomless anal sex in this high-risk population. The present study examined correlates of condomless anal sex among 201 MSM recruited in Tijuana, Mexico, with a focus on the role of public sex venues in relation to sexual risk behavior. Eligibility requirements were: biologically male, 18 years of age or older, resident of Tijuana, and self-reported anal or oral sex with a male partner in the past year. Participants completed an interviewer-administered, demographic and psychosocial survey, and were tested for HIV and syphilis. A hierarchical multiple linear regression model was tested to identify correlates of condomless anal sex. Thirty-eight percent of participants (*N =* 76) reported condomless anal sex with a male partner in the past 2 months. Higher levels of condomless anal sex were associated with higher levels of depressive symptoms, greater sexual compulsivity, and more frequent seeking out of sex partners in a public venue in the past 2 months. In view of these findings, we recommend the development of multi-level, “combination” interventions, which in the Mexican context should include enhanced condom promotion and distribution, improved availability and access to mental health treatment and counseling services, and expanded HIV/STI testing in public venues.

## Introduction

Research into the correlates of sexual risk behavior among men who have sex with men (MSM) has a decades-long history, as researchers have sought to identify and understand changes in sexual risk behaviors associated with sociocultural and political change as well as with technological and medical advances [1]. The majority of studies have been conducted in developed countries and thus have limited application in low- to middle-income countries (LMIC), where stigma, discrimination, intolerance, and even criminalization of same sex behavior are often more severe [2, 3]; hence, the behavioral, psychological, and contextual correlates of sexual risk behavior are likely to differ.

Mexico ranks eleventh in population among world countries, with an estimated 124 million inhabitants [4]. Although in the past two decades it has undergone rapid social, cultural, economic, and technological change, Mexico continues to meet the criteria for a LMIC. Prevalent social mores in Mexico surrounding sexuality and masculinity tend to be highly traditional [5–7], and stigma associated with homosexuality continues to affect how gay men are treated, with experiences of homophobia and discrimination against MSM being common [8]. In addition, MSM in Mexico have disproportionately high rates of HIV infection (16.9% compared to 0.23% among reproductive age adults)[9], which has contributed to further stigma and discrimination [8, 10].

Research in developed countries and in LMIC has identified a range of psychosocial factors associated with sexual risk behavior among MSM, including psychological vulnerabilities (e.g., depressive symptoms, internalized homophobia, sexual compulsivity), substance use (drugs, alcohol), and impoverished personal and social characteristics, such as low educational attainment and high unemployment [11–15]. The venues where MSM meet their sexual partners have also been identified as a contextual factor that influences sexual risk behavior [16, 17].

Several studies of MSM in high-income countries, including the US and Western Europe, have documented links between depressive symptoms and risky sexual behavior [18–21], including receptive and insertive anal intercourse. In studies of MSM in LMIC, including India and China, clinically-significant depressive symptoms have been identified as a predictor of unprotected anal sex with male partners [12, 15, 22], and a greater number of male partners [23].

Internalized homophobia, which refers to dissatisfaction with being homosexual [24], has also been associated in the US and Europe with sexual risk behavior among MSM [25–28, 29]. In LMIC, including Uganda, Nigeria, and South Africa, internalized homophobia among MSM was associated with unprotected anal intercourse with male partners and HIV infection [3, 11, 30, 31].

Numerous studies have also documented the association between sexual compulsivity and sexual risktaking among MSM in the US and other developed countries [32–36]. In LMIC, at least two studies have reported a relationship between sexual compulsivity and sexual risk behavior among MSM [37, 38].

Studies that link substance use to high levels of risky sexual behavior among MSM in the US are plentiful [19, 36, 39–43]. In LMIC, a strong association between alcohol use and unprotected anal sex among MSM has been well documented [44–48]. In a study of MSM in Tijuana, Mexico, the use of illicit drugs before or during sex was associated with more unprotected anal sex [16].

The relationship between personal and social characteristics of MSM and condomless anal sex both in developed countries and in LMIC has yielded consistent findings; in short, the more impoverished the individual, the more sexual risk behavior [19, 36, 49, 50]. In studies of MSM in LMIC where same sex behavior is highly stigmatized, including Ghana, India, Thailand, and China, condomless anal sex with men has been associated with younger age, less education, HIV-positive serostatus, and cohabitation with a partner of either sex [15, 48, 51, 52, 53].

Although the frequenting by MSM of public venues for sex with other men has received limited attention, studies in the US and China have documented an association between seeking sex partners in public venues and higher levels of unprotected anal sex [17, 46, 54–57, 58].

The present study examined correlates of condomless anal sex among MSM in Tijuana, Mexico. We focused on condomless anal sex with male partners since it is the primary mode of HIV transmission among MSM in Mexico [59]. Based on previous literature, including studies that report high levels of anti-gay stigma and discrimination in Mexico [6, 7], we hypothesized that greater experiences of condomless anal sex with male partners would be associated with psychological risk factors (e.g., internalized homophobia, depression), substance use, and impoverished personal and social characteristics. We also examined whether the propensity to seek out sex partners in public venues predicts greater experiences of condomless anal sex above and beyond the contribution made by the factors just mentioned. Because of the lack of information on the use of public sex venues by MSM in LMIC, we also examined descriptive data on the frequency and types of sexual activities and substance use that occurred in public settings. Identifying correlates of condomless anal sex among MSM in Tijuana has the potential to inform the development of sexual risk reduction programs for MSM in the border region and elsewhere in Mexico, as well as in other LMIC.

The current study differs from a previous study based on these data where we reported a relationship between number of syndemic factors (i.e., co-occurring psychosocial factors), and sexual risk behavior, as well as the attenuation effect of “outness” about having sex with men [60]. Although our definition of syndemic conditions included some of the same psychosocial variables that were used in the current analyses, our previous work did not address the relationship between public venues and sexual risk behavior, while taking into account the effects of psychosocial and substance use factors.

## Methods

### Participants

These analyses used data gathered from a sample of 201 MSM who were recruited for a study of HIV prevalence and related risk factors among MSM in Tijuana, Mexico during 2012–2013. To be eligible, participants had to: be biologically male, at least 18 years of age, and a resident of Tijuana; and report having anal or oral sex with a male partner in the past year. The recruitment method used was respondent-driven sampling (RDS). RDS uses peer referral, a dual incentive system, and a mathematical approach with probability weights to adjust for non-random sampling biases [61]. Six seeds were selected to initiate RDS recruitment, and four more were eventually added to bolster recruitment. Seeds were diverse with respect to age, ethnicity, socioeconomic status, and sexual orientation. Each received three numbered coupons to refer MSM in their social network to the study, and an incentive of $5 US was paid for each referral that resulted in enrollment of an additional participant [62]. All but one seed were productive; the nine productive seeds resulted in an average of 19 referrals (SD = 34.9; Median = 6.5; range = 1 to 113).

In addition to receiving his own set of coupons to refer further potential participants, each peer recruit who enrolled was paid $20 US for a two-hour, face-to-face interview using a standardized survey at the offices of the Agencia Familiar Binacional (AFABI), a community-based HIV services organization located in central Tijuana. A face-to-face interview was chosen over computer-assisted self-interviewing because the research team wanted direct feedback from participants on the survey, which was to be used in a future, large-scale study of this risk population. Interview topics included socio-demographics, drug and alcohol use, mood, sexual risk behavior, STI history, and HIV/STI knowledge as well as childhood and adulthood experiences of stigma, homophobia, discrimination, and violence. After receiving pre-test counseling, participants also provided a finger-stick blood sample for rapid testing for HIV (Advanced Quality HIV Test Kits, Intec Products, Inc., Xiamen, China) and syphilis (Advanced Quality Anti-TP, Intec Products, Inc., Xiamen, China). Post-test counseling was then provided, as was antibiotic treatment for participants who received a positive syphilis result. Positive HIV results were confirmed through an additional (venous) blood sample that was sent to the San Diego County Public Health Laboratories (SDCPHL) for confirmation via immunofluorescence assay.

### Measures

#### Number of condomless anal sex acts with male partners

MSM were asked to report the number of times during the past 2 months that they (a) engaged in anal sex with a male partner and (b) used a condom for anal sex with a male partner. A summary variable, total number of condomless anal sex acts with male partners in the past 2 months, was calculated by subtracting (b) from (a). This was defined as the dependent variable in the analyses.

#### Personal and social characteristics

Participants reported their age, number of years of formal education, marital status, sexual orientation, employment status, income, living situation, HIV serostatus, and whether they self-identify as a sex worker.

#### Substance use factors

For each item in a list of 13 illicit drugs (e.g., marijuana, cocaine, methamphetamine), participants were asked if they had used that drug in the past month. A dichotomous variable was calculated to represent use of at least one illicit drug in the past month (1 = yes, 0 = no). Severity of alcohol use during the last year was measured by the Alcohol Use Disorders Test (AUDIT-10) [63, 64].

#### Psychological risk factors

Depressed mood was measured using the 21-item Beck Depression Inventory (BDI), which has undergone extensive reliability and validation studies (alpha = 0.81) [65, 66]. The 10-item Sexual Compulsivity Scale was used to measure “obsessive preoccupations with sexual acts and encounters” (alpha = 0.92) [67, p.588]. The 9-item internalized homophobia (IHP) scale [24, 68, 69] measures “dissatisfaction with being homosexual and desire to become heterosexual” (alpha = 0.89) [24, p. 2]. Mean scores were computed for sexual compulsivity and internalized homophobia, and a summary score was computed for Beck Depression.

#### Venues used by participants to seek out male sex partners

Participants were presented with a list of commercial and public venues where men can go to meet male sexual partners. Commercial venues included the following: bathhouse, sauna, adult movie theatre, dark room, bar, night club or disco, café. A public venue was defined as a park, public restroom, bus station, canyon, alley, or open space. For each venue, participants were asked, “In the past two months, did you go to a (venue) with the intention of meeting male sex partners?” (1 = yes, 0 = no). Two dichotomously-scored variables were created to represent the locations where MSM sought out male sex partners in the past 2 months: public venue (1 = yes, 0 = no); commercial venue (1 = yes, 0 = no).

### Statistical analyses

We first examined frequency distributions for all variables. Two that had positively-skewed distributions (i.e., “total number of unprotected anal sex acts” and “total number of sex partners in the past 2 months”) were log-10 transformed. A hierarchical multiple linear regression model predicting number of condomless anal sex acts with male partners was then tested. In a previous study using the same data, homphily and clustering by respondent, recruitment chain, geographic area and common-recruiter cluster levels were evaluated using one-way ANOVA [62]. We found no effect of sampling method (i.e., clustering) on these data; thus, unweighted data were used in the present analyses. The dependent variable (total number of condomless anal sex acts) was regressed on four blocks of variables: personal and social characteristics (Step 1), substance use factors (Step 2), psychological risk factors (Step 3), and venues used to meet male sex partners (Step 4).

### Ethical statement

This protocol was reviewed and approved by the UCSD Human Research Protections Program and the Ethics Committee of Universidad Autónoma de Baja California. All participants provided written informed consent. All procedures were conducted in accordance with the Helsinki Declaration.

## Results

### Participant characteristics

Approximately 97% of participants self-identified as gay or bisexual (63.5% and 33.0%, respectively). The average age was 29.7 (SD = 8.7, median *=* 28, range 18–65), 49% had a high school education or greater, 82% were never married, 18% had a male spouse or steady partner, 60% were employed, and 54.5% reported an average monthly income of > 3500 pesos (> $197 USD). The majority lived alone or with another person who was not a sex partner (33.7% and 39.2%, respectively). Twenty-four percent (n = 48) reported having a female sex partner in the past 2 months. Among those with a female partner, 58% (n = 28) reported having anal sex with their female partner in this time period. Two MSM were previously diagnosed HIV-positive and 33 were newly diagnosed in this study, resulting in a HIV prevalence of 17.4% (35/201)(see Table 1).

**Table 1:** Baseline characteristics of men who have sex with men (MSM) in Tijuana, Mexico (*N =* 201).

	N	Percent^a^
**Personal and social characteristics**		
Age (Mean, SD)	29.7	8.7
Sexual orientation		
Gay	127	63.5
Bisexual	66	33.0
Heterosexual	7	3.5
Living arrangement (lives with male spouse or steady vs. other)	35	17.6
Education (high school or more vs. less than high school)	97	48.3
Employment (employed vs. not employed)	120	60.0
Marital status (never married vs. other)	157	80.1
Average monthly income (> 3500 pesos vs. ≤3500 pesos)	105	54.5
Had a previous HIV-positive test result	2	1.0
HIV serostatus via rapid test result (HIV-positive vs. HIV-negative)	35	17.4
Self-identifies as a sex worker	28	14.6
Had female sexual partner^b^	48	24.0
**Psychological risk factors**		
Sexual compulsivity (Mean, SD)	1.74	0.62
Depressive symptoms (Mean,SD)	10.6	7.8
Internalized homophobia (Mean, SD)	2.1	0.72
**Sexual risk behavior**		
Number of steady or regular partners^b^ (Mean, SD)	1.4	2.4
Number of casual sex partners^b^ (Mean, SD)	3.0	5.8
Number of anonymous partners^b^(no money exchanged)(Mean, SD)	1.7	5.5
Number of exchange for sex partners^b^(Mean, SD)	2.4	8.0
Total number of sex partners^b^ (Mean, SD)	7.1	13.7
Number of anal sex acts with male partners in past 2 months (Mean, SD)	18.8	22.4
Number of unprotected anal sex acts with male partners^b^ (Mean, SD)	6.4	13.9
Number of vaginal sex acts with a woman^b^ (Mean, SD) (n = 45)	14.8	21.3
Number of unprotected vaginal sex acts with a woman^b^ (Mean, SD) (n = 45)	9.6	21.2
Number of anal sex acts with a women^b^ (Mean, SD) (n = 46)	8.7	20.6
Number of unprotected anal sex acts with a woman^b^ (Mean, SD) (n = 46)	6.0	19.5
**Substance use**		
Used illicit drugs^c^	83	41.3
AUDIT-10^d^	7.9	8.1
Hazardous drinking (AUDIT-10 ≥ 8)^d^	90	45.0
Number of different drugs used (lifetime) (Mean, SD)	1.7	2.0
Injected drugs^b^	4	2.0
Used alcohol before or during sex^b^	83	41.3
Used drugs before or during sex^b^	24	12.6
**Partner seeking venues**		
Sought out sex partners in public venue^b^	46	22.9
Sought out sex partners in commercial venue^b^	93	46.3

^a^Based on valid percent;

^b^In past 2 months;

^c^In the past month;

^d^In past year

### Use of public venues by MSM to meet male sex partners in Tijuana

Twenty-three percent (*N =* 46) reported going to a public venue in the past 2 months with the intention of meeting male sex partners. Eighty-five percent of MSM who sought out male sex partners in a public venue succeeded in meeting one there. The average number of sex partners that participants reported meeting in a public venue in the past 2 months was 7.6 (SD = 11.6, range 1 to 50). Approximately 60% of participants reported that they had not only met their sex partner in the public venue but also had sex with him there. Such men reported engaging in the following sex acts at the venue: insertive anal sex without a condom (57.9%), receptive anal sex without a condom (52.6%), received oral sex without a condom (73.7%), gave oral sex without a condom (52.6%), group sex with other men (35.3%). Twenty-eight percent of men who had sex in a public venue reported being drunk during their sexual encounter, and 33% reported being high on drugs. Among those who reported using drugs in connection with sex in a public venue, the most frequently used drug was methamphetamine.

### Hierarchical linear regression predicting condomless anal sex with male partners

In Step 1, personal and social characteristics accounted for less than one percent of the variance in the dependent variable (DV) (R^2^ Δ = .006). No variables were statistically significant at this step. In Step 2, substance use factors contributed an additional 1% of variance (R^2^ Δ = .007). None of the substance use variables yielded statistical significance. In Step 3, psychological factors accounted for an additional 10% of the variance in condomless anal sex acts (R^2^ Δ = .097). MSM who scored higher on sexual compulsivity reported more acts of condomless anal sex with male partners in the past 2 months (beta = 0.197, p < .05). Also, more condomless anal sex acts with male partners in the past 2 months was associated with higher scores on depressive symptoms (beta = .208, p < .01). Internalized homophobia was not associated with number of condomless anal sex acts. In the fourth and final step, two venue types that participants reported using to meet male sex partners accounted for approximately 3% of the variance in condomless anal sex (R^2^ Δ = .028). As hypothesized, MSM who sought out male sex partners in public venues reported significantly more acts of condomless anal sex with male partners in the past 2 months (beta = .182, p < .05). Thus, in the final model, more acts of condomless anal sex with male partners in the past 2 months were independently associated with greater sexual compulsivity, more depressive symptoms, and seeking out sex partners in public venues (see Table 2). Because almost one-quarter (24%) of our sample reported having sex with both men and women in the past two months (MSMW), we repeated our hierarchical regression analysis with 48 MSMW cases excluded. The substantive findings did not change. In a separate sensitivity analysis, we examined the potentially confounding effects of HIV-positive (HIV+) serostatus on the outcome by excluding the 35 HIV+ MSM (33 newly diagnosed HIV+ and 2 previously identified HIV+) from the regression analyses. With HIV+ MSM excluded, depressive symptoms were no longer significant in relation to the outcome; however, the association between condomless anal sex and all of the other correlates remained unchanged. The loss of significance for depressive symptoms is most likely attributable to reduced statistical power associated with the smaller sample size.

**Table 2:** Number of condomless anal sex acts with male partners in the past 2 months regressed on personal and social characteristics (Step 1), substance use factors (Step 2), psychological risk factors (Step 3) and venues used to meet sex partners (Step 4) among men who have sex with men in Tijuana, Mexico (*N* = 197)^a^.

	Step 1		Step 2		Step 3		Step 4	
	beta	sr^2^	beta	sr^2^	beta	sr^2^	beta	sr^2^
Sexual orientation (gay vs other)	.047	.002	.070	.004	.052	.002	.083	.005
Age (in years)	-.036	.001	-.035	.001	-.077	.005	-.058	.003
Living arrangement (living with male spouse/steady partner vs other)	-.050	.002	-.047	.002	-.032	.001	-.023	.001
AUDIT-10^b^			.017	.000	-.062	.003	-.067	.004
Use of illicit drugs^c^ (y/n)			.084	.006	.058	.003	.038	.001
Beck Depression					.208**	.033	.221**	.038
Sexual compulsivity					.197*	.029	.174*	.022
Internalized homophobia					-.077	.004	-.065	.003
Sought out sex partner(s) in public venue^d^ (y/n)							.182*	.028
Sought out sex partner(s) in commercial venue (e.g.,bathhouse/adult theater/ bar/nightclub^d^ (y/n)							-.053	.002
Constant	.390**		.326*		.138		.073	
R^2^	.006		.013		.110		.139	
R^2^ change	.006		.007		.097***		.028*	
F (df)	0.37 (3,193)		0.50 (5,191)		2.92** (8,188)		2.99**(10,186)	

beta = standardized regression coefficient;

^‡^ p < .10

*p < .05;

**p < .01;

***p < .001 (2-tailed tests)

^a^ Four cases missing data;

^b^In the past year;

^c^In past month;

^d^In the past 2 months

sr^2^ = the proportion of variance explained in the dependent variable (DV) by a specific independent variable

R^2^ = proportion of variance in the DV explained by the regression model

R^2^ Δ = proportion of variance in the DV accounted for by the block of variables at that step in the regression

## Discussion

In our RDS-recruited sample of MSM in Tijuana, Mexico, 38% of participants reported condomless anal sex with a male partner in the past 2 months. Although direct comparisons are made difficult by varying time frames, this estimate falls within the range of estimates for condomless anal sex reported for MSM in other LMIC [44, 47, 48, 70, 71]. Higher levels of condomless anal sex were associated with more depressive symptoms, higher sexual compulsivity scores, and partner-seeking in public venues. As hypothesized, seeking partners in public venues was significantly associated with condomless anal sex, even after controlling for psychological risk factors, substance use, and personal and social characteristics.

Psychological risk factors accounted for the largest proportion of variance in sexual risk behavior. Within the psychological domain, depressive symptoms had the strongest association with condomless anal sex. The link between depressive symptoms and sexual risktaking has been documented in previous studies of MSM [19, 21, 22]; however, the causal pathways underlying this relationship are not fully understood. Potential pathways include substance use, escape or avoidant coping, low self-efficacy for condom negotiation, impaired decision-making, and impaired ability to plan for safer sex encounters [72–76]. Future studies of MSM in Mexico should examine these pathways as well as others that may be unique to the local culture and risk environments (e.g., social isolation, loneliness, fewer gay community attachments).

Based on clinical cutpoints for the BDI total score [77], 20% of our sample met criteria for moderate to severe depression (i.e., BDI score ≥ 17). The association between clinically- significant depressive symptoms and condomless anal sex puts depressed MSM at higher risk for HIV/STI infection [19]. MSM whose depressive symptoms tend to worsen in the aftermath of episodes of sexual risk behavior may also be at increased risk for suicidal thoughts and self-harm [78]. Local governments and federal agencies in Mexico would be well advised to scale up treatment services that are responsive to the mental health needs of MSM. Being MSM or gay in a culture that stigmatizes and discriminates against same-gender sexual behaviors may be at the root of depressive symptomatology. Future research should seek to identify social, psychological, economic, and cultural antecedents of depressive symptoms.

Higher sexual compulsivity scores were also associated with more condomless anal sex.

This finding has been documented with consistency in samples of gay and bisexual men as well as among male clients of female sex workers in Tijuana [33, 34, 79]. Previous studies have also found that sexual compulsivity co-occurs with other psychosocial and behavioral risk factors, including illicit drug use, substance use before or during sex, polydrug use, depressive symptoms, and childhood sexual abuse [34, 80–82]. In a study of MSM in a rural region of the US, Satinsky et al. [83] found a positive association between sexual compulsivity and partner-seeking in highly sexualized venues, including public cruising spots. As suggested by Parsons and Halkitis [57], seeking partners in public venues requires little planning and thus may be an ideal location for acting upon compulsive sexual urges. Overall, our data support the conclusion that sexual risk reduction interventions for MSM in Mexico should address sexual compulsivity as a risk factor that may co-occur with other psychosocial risk factors [34]. Also, as others have noted, sexually compulsive behavior varies in intensity between individuals, ranging from mildly problematic to serious disorders meeting DSM criteria [84]. Our data indicate the need for health care providers in public and private clinics in Mexico to screen for sexually compulsive behaviors by routinely asking patients if they have experienced sexual thoughts, urges or behaviors that cause them personal distress or that negatively affect aspects of their life, such as job, health, relationships [85]. Psychological treatments, including behavioral and cognitive techniques, should be made available to individuals who report distress or exhibit clinically significant levels of sexually compulsive behavior [32, 84].

This study also found a strong relationship between seeking male partners in public venues and condomless anal sex in the past 2 months. Previous research conducted mostly in the US indicates that characteristics associated with partner-seeking in public venues include substance use, sexual compulsivity, psychological distress, sex trading, HIV-positive serostatus, and degree of “outness” [17, 54, 56, 57]. To date, little is known about the characteristics of MSM who seek partners in public venues in Tijuana, Mexico. Because many of these venues are in secluded outdoor areas and thus not easily accessed by outreach workers, it is questionable whether HIV prevention programs can be effectively implemented in them, whether in Tijuana or elsewhere in Mexico. Programs are thus needed to reach MSM in public areas that are unfamiliar and sometimes concerning to outreach workers. One possible approach would be to train peer outreach workers who are familiar with and frequent these public areas to distribute information on free HIV testing and other HIV/STI prevention services to persons they encounter. Moreover, since sex in public places has been associated with less condom use for anal sex and greater use of substances among MSM [16, 46], future studies should examine the acceptability of antiretroviral (ARV) pre-exposure prophylaxis (PrEP) products (oral pills or topic gels) as an alternative user-controlled HIV prevention method. Although PrEP is not currently available in Mexico, studies that examine acceptability and identify barriers to uptake and adherence in this high-risk population are warranted.

Future research should attempt to identify the characteristics of MSM who seek out sex partners in public venues and detail the frequency and types of sexual activities that occur in these settings. Findings from the present study indicate that most sexual behavior in public venues was high-risk (58% reported insertive anal sex without a condom; 53% reported receptive anal sex without a condom). A significant proportion of such behavior also involved stimulant use, alcohol intoxication, or both.

Overall, the findings from this study are potentially useful in terms of developing a screening tool to identify MSM who are at high risk for engaging in condomless anal sex (e.g., elevated depressive symptoms, sexually compulsive, seek partners in public venues) so that interventions, including sexual risk reduction counseling and regular HIV testing, can be recommended to them by health care providers. Despite the potential usefulness of these findings for HIV prevention and intervention, we call for future research to identify the full range of social, legal, political, and other factors that influence the decision to seek out partners in a public venue. For example, some researchers have called for wider investigation of the role of policing practices in relation to partner seeking and sexual risk behaviors in public venues [86, 87]. This study has several limitations. Because it was a single-site study, our findings cannot be generalized to MSM in the rest of Mexico or in other LMIC. Also, the cross-sectional design does not permit us to assess causality in the relationships between predictor variables and condomless anal sex. The retrospective and self-reported nature of our data also has the potential to bias this study’s findings. In particular, we believe that drug use may have been underreported, especially when compared with reports of drug use among Latino MSM in the adjacent county of San Diego, California [88]. Also, this study did not ask participants about their use of the internet for meeting male sex partners. This is a notable shortcoming, since other studies have shown that MSM who meet partners over the internet are more likely to engage in sexual risk behavior than those who do not meet partners this way [89, 90]. Moreover, we did not ask whether participants used private venues to meet partners, such as invitation-only sex parties, which have been linked to high-risk sexual behavior [91], nor did we inquire about participants’ sexual activities in other non-public venues such as homes or hotel rooms [17]. As previously mentioned, research has documented the association between a number of personal and social characteristics and condomless anal sex among MSM; however, only a limited number of these variables could be included in our regression model due to sample size. Future studies should aim for larger sample sizes so that a wider range of these variables (e.g., education, income, STI history) can be examined in relation to condomless anal sex. The smallness of our sample also prevented us from conducting separate analyses for MSM and MSMW that might otherwise have yielded interesting results, since previous research has shown significant differences between the two groups. For example, MSMW in LMIC have higher rates of inconsistent use of condoms with male partners, greater overall use of alcohol and illicit drugs, and higher likelihood of sex trading than men who have sex only with men (MSM) in the same countries [92, 93]. Future studies could examine the correlates of sexual risk behavior separately for MSM and MSMW, particularly in LMIC where gay stigma and discrimination are known to influence sexual identity and practices [94]. Moreover, the high rate of sex with women (24%) and the high levels of unprotected vaginal and anal sex with female partners among MSMW calls for more research and development of targeted interventions for this subpopulation of men in LMIC. Finally, this study did not include transgender persons, a group that is at high risk for HIV/STIs yet remains understudied around the globe [8, 95]. Enhanced understanding of the correlates of sexual risk behavior among transgender persons will help to inform the development of much needed, tailored prevention interventions and treatment services.

## Conclusions

The finding that more than one-third of MSM in Tijuana reported condomless anal sex in the past 2 months should be a matter of serious concern to public health authorities, given the relationship between this high-risk behavior and increased risk for contracting HIV/STIs [96]. Clearly, evidence-based approaches are needed to target the behavioral and psychological processes that influence high-risk sexual behavior in MSM. Currently, few such approaches are available in Mexico or in other LMIC [97]. To maximize their effectiveness, prevention programs should be culturally sensitive and address multiple and often co-occurring psychosocial factors [15, 98]. The confluence of psychological risk factors prevalent among MSM (i.e., depression, sexual compulsivity) with the propensity to seek out high-risk public venues for sex, calls for multi-level, “combination” interventions designed to reduce sexual risk behavior. Mayer et al. [99] refer to “prevention packages with culturally-tailored components” (p.6) that in the Mexican context would ideally include enhanced promotion of condom use and distribution of condoms, improved availability of and access to mental health treatment and counseling services, and expanded HIV/STI testing in public venues. Such interventions would also ideally address prevailing social conditions in Mexico, including poverty, homophobia, discrimination and violence against MSM [100–102].

## Supporting information

S1 Data File Data for study on correlates of anal sex among MSM in Tijuana, Mexico. (SAV)

## References

[1] Jerome RC, Woods WJ, Moskowitz JT, Carrico AW. The psychological context of sexual compulsivity among men who have sex with men. AIDS Behav. 2016;20(2):273–80. doi: 10.1007/s10461-015-1083-1 25957856 10.1007/s10461-015-1083-1 PMC5482006

[2] Altman D, Aggleton P, Williams M, Kong T, Reddy V, Harrad D, et al Men who have sex with men: stigma and discrimination. Lancet. 2012;380(9839):439–45. doi: 10.1016/S0140-6736(12)60920-9 22819652 10.1016/S0140-6736(12)60920-9

[3] Ross MW, Kajubi P, Mandel JS, McFarland W, Raymond HF. Internalized homonegativity/homophobia is associated with HIV-risk behaviours among Ugandan gay and bisexual men. Int J STD AIDS. 2013;24(5):409–13. doi: 10.1177/0956462412472793 23970711 10.1177/0956462412472793

[4] Mexico population World Population Review. http://worldpopulationreview.com/countries/mexico-population. Accessed on 16 Dec 2016.

[5] Maternowska MC, Withers M, Brindis C. Gender, Masculinity and migration: Mexican men and reproductive health in the California context. Cult Health Sex. 2014;16(8):989–1002. doi: 10.1080/13691058.2014.920529 24939475 10.1080/13691058.2014.920529

[6] Steffens MC, Jonas KJ, Denger L. Male Role Endorsement Explains Negative Attitudes Toward Lesbians and Gay Men Among Students in Mexico More Than in Germany. J Sex Res. 2015;52(8):898–911. doi: 10.1080/00224499.2014.966047 25369554 10.1080/00224499.2014.966047

[7] Verduzco IL. Barriers to Sexual Expression and Safe Sex Among Mexican Gay Men: A Qualitative Approach. Am J Mens Health. 2014;1–15. 10.1177/1557988314561490 25504646

[8] Cuadra-Hernandez SM, Zarco-Mera A, Infante-Xibille C, Caballero-Garcia M. The organization of key populations connected to HIV transmission: an intervention to abate stigma; Mexico, 2005–2009. Salud colectiva. 2012;8(2):191–204. doi: 10.1590/S1851-82652012000200007 23995546 10.18294/sc.2012.158

[9] Secretaria de Salud, Centro Nacional para la Prevención y el Control del VIH y el SIDA (CENSIDA). Informe nacional de avances en la respuesta al vih y el sida: Mexico 2015. 2015. http://www.unaids.org/sites/default/files/country/documents/MEX_narrative_report_2015.pdf. Accessed on 24 Aug 2017.

[10] Bautista-Arredondo S, Colchero MA, Romero M, Conde-Glez CJ, Sosa-Rubi SG. Is the HIV Epidemic Stable among MSM in Mexico? HIV Prevalence and Risk Behavior Results from a Nationally Representative Survey among Men Who Have Sex with Men. PLoS ONE. 2013;8(9):e72616 doi: 10.1371/journal.pone.0072616 24039786 10.1371/journal.pone.0072616 PMC3764146

[11] Adebajo SB, Eluwa GI, Allman D, Myers T, Ahonsi BA. Prevalence of internalized homophobia and HIV associated risks among men who have sex with men in Nigeria. AIDS Care. 2012;16(4):21–8. 23444540

[12] Mimiaga MJ, Biello KB, Sivasubramanian M, Mayer KH, Anand VR, Safren SA. Psychosocial risk factors for HIV sexual risk among Indian men who have sex with men. AIDS Care. 2013;25(9):1109–13. doi: 10.1080/09540121.2012.749340 23339580 10.1080/09540121.2012.749340 PMC3708996

[13] Mustanski B, Garofalo R, Herrick A, Donenberg G. Psychosocial health problems increase risk for HIV among urban young men who have sex with men: preliminary evidence of a syndemic in need of attention. Ann Behav Med. 2007;34(1):37–45. 17688395 10.1080/08836610701495268 PMC2219199

[14] Stall R, Mills TC, Williamson J, Hart T, Greenwood G, Paul J, et al Association of co-occurring psychosocial health problems and increased vulnerability to HIV/AIDS among urban men who have sex with men. Am J Public Health. 2003;93(6):939–42. 12773359 10.2105/ajph.93.6.939 PMC1447874

[15] Thomas B, Mimiaga MJ, Menon S, Chandrasekaran V, Murugesan P, Swaminathan S, et al Unseen and unheard: predictors of sexual risk behavior and HIV infection among men who have sex with men in Chennai, India. AIDS Educ Prev. 2009;21(4):372–83. doi: 10.1521/aeap.2009.21.4.372 19670971 10.1521/aeap.2009.21.4.372 PMC3623672

[16] Barrón-Limón S, Semple SD, Strathdee SA, Lozada R, Vargas-Ojeda A, Patterson TL. Correlates of unprotected anal sex among men who have sex with men in Tijuana, Mexico. BMC Public Health. 2012. 10.1186/1471-2458-12-433 PMC3432613 22694837

[17] Semple SJ, Strathdee SA, Zians J, Patterson TL. Factors associated with sex in the context of methamphetamine use in different sexual venues among HIV-positive men who have sex with men. BMC Public Health. 2010;10:178 doi: 10.1186/1471-2458-10-178 20359362 10.1186/1471-2458-10-178 PMC2858118

[18] De Santis JP, Colin JM, Provencio Vasqueuz E, McCain GC. The relationship of depressive symptoms, self-esteem, and sexual behaviors in a predominately Hispanic sample of men who have sex with men. Am J Mens Health. 2008;2(4):314–21. doi: 10.1177/1557988307312883 19477795 10.1177/1557988307312883

[19] Fendrich M, Avci O, Johnson TP, Mackesy-Amiti ME. Depression, substance use and HIV risk in a probability sample of men who have sex with men. Addict Behav. 2013;38(3):1715–8. doi: 10.1016/j.addbeh.2012.09.005 23254224 10.1016/j.addbeh.2012.09.005 PMC3619198

[20] Poppen PJ, Reisen CA, Zea MC, Bianchi FT, Echeverry JJ. Predictors of unprotected anal intercourse among HIV-positive Latino gay and bisexual men. AIDS Behav. 2004;8(4):379–89. doi: 10.1007/s10461-004-7322-5 15690111 10.1007/s10461-004-7322-5

[21] Wim VB, Christiana N, Marie L. Syndemic and other risk factors for unprotected anal intercourse among an online sample of Belgian HIV negative men who have sex with men. AIDS Behav. 2014;18(1):50–8. doi: 10.1007/s10461-013-0516-y 23681697 10.1007/s10461-013-0516-y

[22] Jie W, Ciyong L, Xueqing D, Hui W, Lingyao H. A syndemic of psychosocial problems places the MSM (men who have sex with men) population at greater risk of HIV infection. PLoS One. 2012;7(3). 10.1371/journal.pone.0032312 PMC3316524 22479319

[23] Safren SA, Thomas BE, Mimiaga MJ, Chandrasekaran V, Menon S, Swaminathan S, et al Depressive symptoms and human immunodeficiency virus risk behavior among men who have sex with men in Chennai, India. Psychol Health Med. 2009;14(6):705–15. doi: 10.1080/13548500903334754 20183543 10.1080/13548500903334754 PMC3640638

[24] Herek GM, Cogan JC, Gillis JR, Glunt EK. Correlates of internalized homophobia in a community sample of lesbians and gay men. J Gay Lesbian Med Assoc. 1997;2(17–25).

[25] Meyer I, Dean L. Internalized homophobia, intimacy, and sexual behaviour among gay and bisexual men In: (Ed.) G H, editor. Stigma and sexual orientation. Thousand Oaks, CA: Sage Publications; 1998 p. 160–86.

[26] Ratti R, Bakerman R, Peterson JL. Correlates of high-risk sexual behaviour among Canadian men of South Asian and European origin who have sex with men. AIDS Care. 2000;12:193–202. doi: 10.1080/09540120050001878 10827860 10.1080/09540120050001878

[27] Ross MW, Rosser BRS, Neumaier ER. The Positive Connections Team. The relationship of internalized homonegativity to unsafe sexual behavior in HIV seropositive men who have sex with men. AIDS Educ Prev. 2008;20:547–57. doi: 10.1521/aeap.2008.20.6.547 19072529 10.1521/aeap.2008.20.6.547 PMC2605286

[28] Ross MW, Berg RC, Schmidt AJ, Hospers HJ, Breveglieri M, Furegato M, et al Internalised homonegativity predicts HIV-associated risk behavior in European men who have sex with men in a 38-country cross-sectional study: some public health implications of Homophobia. BMJ. 2013. 10.1136/bmjopen-2012-001928 PMC3586183 23386580

[29] Newcomb ME, Mustanski B. Internalized homophobia and internalizing mental health problems: a meta-analytic review. Clin Psychol Rev. 2010;30(8):1019–29. doi: 10.1016/j.cpr.2010.07.003 20708315 10.1016/j.cpr.2010.07.003

[30] Sandfort TG, Lane T, Dolezal C, Reddy V. Gender Expression and Risk of HIV Infection Among Black South African Men Who Have Sex with Men. AIDS Behav. 2015;19(12):2270–9. doi: 10.1007/s10461-015-1067-1 25869555 10.1007/s10461-015-1067-1 PMC4605836

[31] Ross MW, Rosser BRS, Smolenski DJ. The importance of measuring internalized homophobia /homonegativity. Arch Sex Behav. 2010;39:1207–8. doi: 10.1007/s10508-010-9634-z 20464466 10.1007/s10508-010-9634-z PMC3697840

[32] Coleman E, Horvath KJ, Miner M, Ross MW, Oakes M, Rosser BR, et al Compulsive sexual behavior and risk for unsafe sex among internet using men who have sex with men. Arch Sex Behav. 2010;39(5):1045–53. doi: 10.1007/s10508-009-9507-5 19588239 10.1007/s10508-009-9507-5 PMC3719393

[33] Grov C, Parsons JT, Bimbi DS. Sexual compulsivity and sexual risk in gay and bisexual men. Arch Sex Behav. 2010;39:340–9. 10.1007/s10508-009-9483-9 PMC2890042 19308715

[34] Parsons JT, Grov C, Golub SA. Sexual compulsivity, co-occurring psychosocial health problems, and HIV risk among gay and bisexual men: further evidence of a syndemic. Am J Public Health. 2012;102(1):156–62. doi: 10.2105/AJPH.2011.300284 22095358 10.2105/AJPH.2011.300284 PMC3490563

[35] Semple SJ, Zians J, Grant I, Patterson TL. Sexual compulsivity in a sample of HIV-positive methamphetamine-using gay and bisexual men. AIDS Behav. 2006;10:587–98. doi: 10.1007/s10461-006-9127-1 16755406 10.1007/s10461-006-9127-1

[36] Smolenski DJ, Ross MW, Risser JM, Rosser BR. Sexual compulsivity and high-risk sex among Latino men: the role of internalized homonegativity and gay organizations. AIDS Care. 2009;21(1):42–9. doi: 10.1080/09540120802068803 19085219 10.1080/09540120802068803 PMC3678386

[37] do Amaral ML, Abdo CH, Tavares H, Scanavino Mde T. Personality among sexually compulsive men who practice intentional unsafe sex in Sao Paulo, Brazil. J Sex Med. 2015;12(2):557–66. doi: 10.1111/jsm.12761 25411152 10.1111/jsm.12761

[38] Mimiaga MJ, Biello KB, Robertson AM, Oldenburg CE, Rosenberger JG, O’Cleirigh C, et al High prevalence of multiple syndemic conditions associated with sexual risk behavior and HIV infection among a large sample of Spanish- and Portuguese-speaking men who have sex with men in Latin America. Arch Sex Behav. 2015;44(7):1869–78. doi: 10.1007/s10508-015-0488-2 26159862 10.1007/s10508-015-0488-2

[39] Ayala SB, Bingham T, Kim J, Wheeler DP, Millett GA. Modeling the impact of social discrimination and financial hardship on the sexual risk of HIV among Latino and Black men who have sex with men. Am J Public Health. 2012;102(Suppl 2):S242–9. 22401516 10.2105/AJPH.2011.300641 PMC3477911

[40] Clatts MC, Goldsamt LA, Yi H. Drug and sexual risk in four men who have sex with men populations: evidence for a sustained HIV epidemic in New York City. J Urban Health. 2005;82(1 Suppl 1):9–17. 10.1093/jurban/jti019 PMC2718757 15738325

[41] Fendrich M, Mackesy-Amiti ME, Johnson TP, Pollack LM. Sexual risk behavior and drug use in two Chicago samples of men who have sex with men: 1997 vs. 2002. J Urban Health. 2010;87(3):452–66. doi: 10.1007/s11524-009-9432-x 20217485 10.1007/s11524-009-9432-x PMC2871084

[42] Mackesy-Amiti ME, Fendrich M, Johnson TP. Symptoms of substance dependence and risky sexual behavior in a probability sample of HIV-negative men who have sex with men in Chicago. Drug Alcohol Depend. 2010;110(1–2):38–43. doi: 10.1016/j.drugalcdep.2010.01.016 20219291 10.1016/j.drugalcdep.2010.01.016 PMC2885520

[43] Mustanski B. Moderating effects of age on the alcohol and sexual risk taking association: an online daily diary study of men who have sex with men. AIDS Behav. 2008;12(1):118–26. doi: 10.1007/s10461-007-9335-3 18034298 10.1007/s10461-007-9335-3

[44] Lane T, Shade SB, McIntyre J, Morin SF. Alcohol and sexual risk behavior among men who have sex with men in South African township communities. AIDS Behav. 2008;12(4 Suppl):S78–85. doi: 10.1007/s10461-008-9389-x 18392672 10.1007/s10461-008-9389-x

[45] Mimiaga MJ, Thomas B, Mayer KH, Reisner SL, Menon S, Swaminathan S, et al Alcohol use and HIV sexual risk among MSM in Chennai, India. Int J STD AIDS. 2011;22(3):121–5. doi: 10.1258/ijsa.2009.009059 21464447 10.1258/ijsa.2009.009059 PMC3615984

[46] Tang W, Huan X, Mahapatra T, Tang S, Li J, Yan H, et al Factors associated with unprotected anal intercourse among men who have sex with men: results from a respondent driven sampling survey in Nanjing, China, 2008. AIDS Behav. 2013;17(4):1415–22. doi: 10.1007/s10461-013-0413-4 23334360 10.1007/s10461-013-0413-4

[47] Young SD, Nianogo RA, Chiu CJ, Menacho L, Galea J. Substance use and sexual risk behaviors among Peruvian MSM social media users. AIDS Care. 2016;28(1):112–8. doi: 10.1080/09540121.2015.1069789 26324405 10.1080/09540121.2015.1069789 PMC4713343

[48] Zhang H, Lu H, Pan SW, Xia D, Zhao Y, Xiao Y, et al Correlates of unprotected intercourse: the influence of anal sex position among men who have sex with men in Beijing, China. Arch Sex Behav. 2015;44(2):375–87. doi: 10.1007/s10508-014-0396-x 25548064 10.1007/s10508-014-0396-x

[49] Diaz RM, Ayala G, Bein E. Sexual risk as an outcome of social oppression: data from a probability sample of Latino gay men in three U.S. cities. Cultur Divers Ethnic Minor Psychol. 2004;10(3):255–67. doi: 10.1037/1099-9809.10.3.255 15311978 10.1037/1099-9809.10.3.255

[50] Williams JK, Wyatt GE, Resell J, Peterson J, Asuan-O’Brien A. Psychosocial issues among gay- and non-gay-identifying HIV-seropositive African American and Latino MSM. Cultur Divers Ethnic Minor Psychol. 2004;10(3):268–86. doi: 10.1037/1099-9809.10.3.268 15311979 10.1037/1099-9809.10.3.268

[51] Logie CH, Newman PA, Weaver J, Roungkraphon S, Tepjan S. HIV-related stigma and HIV prevention uptake among young men who have sex with men and transgender women in Thailand. AIDS Patient Care STDS. 2016; 30(2):92–100. doi: 10.1089/apc.2015.0197 26788978 10.1089/apc.2015.0197

[52] Nelson LE, Wilton L, Agyarko-Poku T, Zhang N, Aluoch M, Thach CT, et al The association of HIV stigma and HIV/STD knowledge with sexual risk behaviors among adolescent and adult men who have sex with men in Ghana, West Africa. Res Nurs Health. 2015;38(3):194–206. doi: 10.1002/nur.21650 25809638 10.1002/nur.21650

[53] Wei C, Cheung DH, Yan H, Li J, Shi LE, Raymond HF. The impact of homophobia and HIV stigma on HIV testing among Chinese men who have sex with men: a mediation analysis. J. Acquir Immune Defic Syndr. 2016; 71(1):87–93. 26334742 10.1097/QAI.0000000000000815 PMC4713338

[54] Binson D, Woods WJ, Pollack L, Paul J, Stall R, Catania JA. Differential HIV risk in bathhouses and public cruising areas. Am J Public Health. 2001;91:1482–6. 11527785 10.2105/ajph.91.9.1482 PMC1446808

[55] Diaz RM, Stall RD, Hoff C, Daigle D, Coates TJ. HIV risk among Latino gay men in the Southwestern United States. AIDS Educ Prev. 1996;8(5):415–29. 8911569

[56] Gama A, Abecasis A, Pingarilho M, Mendao L, Martins MO, Barros H, et al Cruising Venues as a Context for HIV Risky Behavior Among Men Who Have Sex With Men. Arch Sex Behav. 2017;46(4):1061–8. doi: 10.1007/s10508-016-0707-5 26987977 10.1007/s10508-016-0707-5

[57] Parsons JT, Halkitis PN. Sexual and drug-using practices of HIV-positive men who frequent public and commercial sex environments. AIDS Care. 2002;14(6):815–26. doi: 10.1080/0954012021000031886 12511214 10.1080/0954012021000031886

[58] Qu L, Wang W, Gao Y, Yang J, Dai J, Wang D, Tao B. A cross-sectional survey of HIV transmission and behavior among men who have sex with men in different areas of inner Mongolia Autonomous Region, China. BMC Public Health. 2016;16(1):1161 doi: 10.1186/s12889-016-3809-z 27846873 10.1186/s12889-016-3809-z PMC5111207

[59] UNAIDS Report on the Global AIDS epidemic 2012. http://files.unaids.org/en/media/unaids/contentassets/documents/epidemiology/2012/gr2012/20121120_UNAIDS_Global_Report_2012_with_annexes_en.pdf. Accessed on 13 Dec 2016.

[60] Pitpitan EV, Smith LR, Goodman-Meza D, Torres K, Semple SJ, Strathdee SA. “Outness” as a moderator of the association between syndemic conditions and HIV risk-taking behavior among men who have sex with men in Tijuana, Mexico. AIDS Behav. 2016;20:431–8. doi: 10.1007/s10461-015-1172-1 26324079 10.1007/s10461-015-1172-1 PMC4755893

[61] Heckathorn DD. Respondent-driven sampling: A new approach to the study of hidden populations. Social Problems. 1997;44(2):174–99.

[62] Pitpitan EV, Goodman-Meza D, Burgos JL, Abramovitz D, Chavarin CV, Torres K, et al Prevalence and correlates of HIV among men who have sex with men in Tijuana, Mexico. J Int AIDS Soc. 2015;18:19304 doi: 10.7448/IAS.18.1.19304 25669423 10.7448/IAS.18.1.19304 PMC4323407

[63] Conigrave KM, Hall WD, Saunders JB. The AUDIT questionnaire: choosing a cut-off score. Alcohol Use Disorder Identification Test. Addiction. 1995;90(10):1349–56. 8616463 10.1046/j.1360-0443.1995.901013496.x

[64] Saunders JB, Aasland OG, Babor TF, de la Fuente JR, Grant M. Development of the Alcohol Use Disorders Identification Test (AUDIT): WHO Collaborative Project on Early Detection of Persons with Harmful Alcohol Consumption—II. Addiction. 1993;88(6):791–804. 8329970 10.1111/j.1360-0443.1993.tb02093.x

[65] Beck AT. Depression: Clinical, experimental and theoretical aspects. New York, NY: Harper & Row; 1967.

[66] Beck AT. Cognitive therapy and emotional disorder. New York, NY: Hoeber; 1976.

[67] Kalichman SC, Rompa D. Sexual sensation seeking and Sexual Compulsivity Scales: reliability, validity, and predicting HIV risk behavior. J Pers Assess. 1995;65(3):586–601. doi: 10.1207/s15327752jpa6503_16 8609589 10.1207/s15327752jpa6503_16

[68] Martin JL, Dean L. Summary of Measures: mental health effects of AIDS on At-risk homosexual men. Division of Socio-medical Sciences, Columbia University, School of Public Health. 1988.

[69] Ross MW, Rosser BR. Measurement and correlates of internalized homophobia: a factor analytic study. J Soc Clin Psychol. 1996;52(1):15–21. 10.1002/(SICI)1097-4679(199601)52:1<15::AID-JCLP2>3.0.CO;2-V 8682906

[70] Tucker A, Liht J, de Swardt G, Jobson G, Rebe K, McIntyre J, et al Homophobic stigma, depression, self-efficacy and unprotected anal intercourse for peri-urban township men who have sex with men in Cape Town, South Africa: a cross-sectional association model. AIDS Care. 2014;26(7):882–9. doi: 10.1080/09540121.2013.859652 24295155 10.1080/09540121.2013.859652

[71] Yi S, Tuot S, Chhoun P, Pal K, Tith K, Brody C. Factors associated with inconsistent condom use among men who have sex with men in Cambodia. PLoS One. 2015;10(8). 10.1371/journal.pone.0136114 PMC4546109 26287731

[72] Alvy LM, McKirnan DJ, Mansergh G, Koblin B, Colfax GN, Flores SA, et al Depression is not associated with sexual risk among men who have sex with men, but is mediated by cognitive escape and self-efficacy. AIDS Behav. 2011;15(6):1171–9. doi: 10.1007/s10461-010-9678-z 20217471 10.1007/s10461-010-9678-z

[73] Bousman CA, Cherner M, Ake C, Letendre S, Atkinson JH, Patterson TL, et al Negative mood and sexual behavior among non-monogamous men who have sex with men in the context of methamphetamine and HIV. Journal of Affective Disorders. 2009;119(1–3):84–91. doi: 10.1016/j.jad.2009.04.006 19419773 10.1016/j.jad.2009.04.006 PMC3051049

[74] McKirnan DJ, Ostrow DG, Hope B. Sex, drugs and escape: a psychological model of HIV-risk sexual behaviours. AIDS Care. 1996;8(6):655–69. doi: 10.1080/09540129650125371 8993716 10.1080/09540129650125371

[75] Safren SA, Traeger L, Skeer MR, O’Cleirigh C, Meade CS, Covahey C, et al Testing a social-cognitive model of HIV transmission risk behaviors in HIV-infected MSM with and without depression. Health Psychol. 2010;29(2):215–21. doi: 10.1037/a0017859 20230095 10.1037/a0017859 PMC2841316

[76] Semple SJ, Patterson TL, Grant I. The sexual negotiation behavior of HIV-positive gay and bisexual men. Journal of Consulting and Clinical Psychology. 2000;68(5):934–7. 11068981

[77] Beck AT, Steer RA. Beck Depression Inventory manual. San Antonio, TX: Psychological Corporation 1987.

[78] Cottler LB, Campbell W, Krishna VA, Cunningham-Williams RM, Abdallah AB. Predictors of high rates of suicidal ideation among drug users. The Journal of nervous and mental disease. 2005;193(7):431–7. doi: 10.1097/01.nmd.0000168245.56563.90 15985836 10.1097/01.nmd.0000168245.56563.90 PMC1350972

[79] Semple SJ, Strathdee SA, Pitpitan EV, Chavarin C, Patterson TL. Behavioral and psychosocial correlates of anal sex among male clients of female sex workers in Tijuana, Mexico. Arch Sex Behav.44(4):1025–33. doi: 10.1007/s10508-015-0514-4 25795530 10.1007/s10508-015-0514-4

[80] Dyer TP, Shoptaw S, Guadamuz TE, Plankey M, Kao U, Ostrow D, et al Application of syndemic theory to black men who have sex with men in the Multicenter AIDS Cohort Study. J Urban Health. 2012;89(4):697–708. doi: 10.1007/s11524-012-9674-x 22383094 10.1007/s11524-012-9674-x PMC3535137

[81] Parsons JT, Kelly BC, Bimbi DS, Muench F, Morgenstern J. Accounting for the social triggers of sexual compulsivity. J Addict Dis. 2007;26(3):5–16. doi: 10.1300/J069v26n03_02 18018804 10.1300/J069v26n03_02

[82] Semple SJ, Zians J, Grant I, Patterson TL. Methamphetamine use, impulsivity, and sexual risk behavior among HIV-positive men who have sex with men. J Addict Dis. 2006;25(4):105–14. doi: 10.1300/J069v25n04_10 17088230 10.1300/J069v25n04_10

[83] Satinsky S, Fisher C, Stupiansky N, Dodge B, Alexander A, Herbenick D, et al Sexual compulsivity among men in a decentralized MSM community of the Midwestern United States. AIDS Patient Care STDs. 2008;22(7):553–60. doi: 10.1089/apc.2007.0255 18479226 10.1089/apc.2007.0255

[84] Rosser BR, Noor SW, Iantaffi A. Normal, problematic and compulsive consumption of sexually explicit media: Clinical findings using the compulsive pornography consumption (CPC) Scale among men who have men with sex. Sex Addict Compulsivity. 2014;21(4):276–304. doi: 10.1080/10720162.2014.959145 26167109 10.1080/10720162.2014.959145 PMC4497958

[85] Compulsive sexual behavior. Defintion and symptoms. http://www.mayoclinic.org/diseases-conditions/compulsive-sexual-behavior/basics/symptoms/con-20020126. Accessed on 26 Aug 2017.

[86] Goldenberg SM, Duff P, Krusi A. Work environments and HIV prevention: a qualitative review and meta-synthesis of sex worker narratives. BMC Public Health. 2015. 10.1186/s12889-015-2491-x PMC4681074 26672756

[87] Shannon K, Strathdee SA, Shoveller J, Rusch M, Kerr T, Tyndall MW. Structural and environmental barriers to condom use negotiation with clients among female sex workers: implications for HIV prevention strategies and policy. Am J Public Health. 2009;99(4):659–65. doi: 10.2105/AJPH.2007.129858 19197086 10.2105/AJPH.2007.129858 PMC2661482

[88] Zellner JA, Martinez-Donate AP, Sanudo F, Fernandez-Cerdeno A, Sipan CL, Hovell MF, et al The interaction of sexual identity and sexual behavior and its influence on HIV risk among Latino men: Results of a community survey in Northern San Diego County, California. Am J Public Health. 2009;99(1):125–32. doi: 10.2105/AJPH.2007.129809 19008512 10.2105/AJPH.2007.129809 PMC2636606

[89] Grov C, Crow T. Attitudes about and HIV risk related to the “most common place” MSM meet their sex partners: comparing men from bathhouses, bars/clubs, and Craigslist.org. AIDS Educ Prev. 2012;24(2):102–16. doi: 10.1521/aeap.2012.24.2.102 22468972 10.1521/aeap.2012.24.2.102 PMC5824633

[90] Lewnard JA, Berrang-Ford L. Internet-based partner selection and risk for unprotected anal intercourse in sexual encounters among men who have sex with men: a meta-analysis of observational studies. Sex Transm Infect. 2014;90(4):290–6. doi: 10.1136/sextrans-2013-051332 24518249 10.1136/sextrans-2013-051332

[91] Grov C, Rendina HJ, Ventuneac A, Parsons JT. Partners met via sex parties present significantly greater odds for condomless anal sex among MSM: an event-level analysis of venues where male partners are met. J Acquir Immune Defic Syndr. 2014;67(5):564–8. doi: 10.1097/QAI.0000000000000343 25226209 10.1097/QAI.0000000000000343 PMC4229438

[92] Ramakrishnan L, Ramanathan S, Chakrapani V, Goswami P, Deshpande S, Yadav D, et al Comparison of sexual risk, HIV/STI prevalence and intervention exposure among men who have sex with men and women (MSMW) and men who have sex with men only (MSMO) in India: Implications for HIV prevention. AIDS Behav. 2015;19:2255–69. doi: 10.1007/s10461-015-1058-2 25893657 10.1007/s10461-015-1058-2 PMC4609307

[93] Wang HY, Xu JJ, Zou HC, Reilly KH, Zhang CM, Yun K, et al Sexual risk behaviors and HIV infection among men who have sex with men and women in China: Evidence from a systematic review and meta-analysis. BioMed research international. 2015. 10.1155/2015/850132 PMC4686633 26779538

[94] Kendall T, Herrera C, Caballero M, Campero L. HIV prevention and men who have sex with women and men in Mexico: findings from a qualitative study with HIV-positive men. Cult Health Sex. 2007;9(5):459–72. doi: 10.1080/13691050601183629 17687672 10.1080/13691050601183629

[95] Peitzmeier SM, Yasin F, Stephenson R, Wirtz AL, Delegchoimbol A, Dorjgotov M, et al Sexual violence against men who have sex with men and transgender women in Mongolia: A mixed-methods study of scope and consequences. PLoS One. 2015;10(10). 10.1371/journal.pone.0139320 PMC4592264 26431311

[96] Baggaley RF, White RG, Boily MC. HIV transmission risk through anal intercourse: systematic review, meta-analysis and implications for HIV prevention. Int J Epidemiol. 2010;39(4):1048–63. doi: 10.1093/ije/dyq057 20406794 10.1093/ije/dyq057 PMC2929353

[97] Baral SD, Wirtz A, Sifakis F, Johns B, Walker D, Beyrer C. The highest attainable standard of evidence (HASTE) for HIV/AIDS interventions: toward a public health approach to defining evidence. Public Health Rep. 2012;127(6):572–84. doi: 10.1177/003335491212700607 23115382 10.1177/003335491212700607 PMC3461350

[98] Tucker A, Liht J, de Swardt G, Jobson G, Rebe K, McIntyre J, et al An exploration into the role of depression and self-efficacy on township men who have sex with men’s ability to engage in safer sexual practices. AIDS Care. 2013;25(10):1227–35. doi: 10.1080/09540121.2013.764383 23387517 10.1080/09540121.2013.764383

[99] Mayer KH, Wheeler DP, Bekker LG, Grinsztejn B, Remien RH, Sandfort TG, et al Overcoming biological, behavioral, and structural vulnerabilities: new directions in research to decrease HIV transmission in men who have sex with men. J Acquir Immune Defic Syndr. 2013(63 Suppl 2):S161–7. 23764630 10.1097/QAI.0b013e318298700e PMC3740716

[100] Orozco-Núñez E, Alcalde-Rabanal JE, Ruiz-Larios JA, Sucilla-Pérez H, Garcia-Cerde R. Discrimination and homophobia associated to the human immunodeficiency virus epidemic. Salud Publica Mex. 2015(57 Suppl 2):190–6. 26545135

[101] Ortiz-Hernandez L, Perez-Salgado D, Tamez-Gonzalez S. Socioeconomic inequality and health in Mexico. Rev Med Inst Mex Seguro Soc. 2015;53(3):336–47. 25984619

[102] Semple SJ, Stockman JK, Goodman-Meza D, Pitpitan EV, Strathdee SA, Chavarin CV, et al Correlates of Sexual Violence Among Men Who Have Sex With Men in Tijuana, Mexico. Arch Sex Behav. 2017;46(4):1011–23. doi: 10.1007/s10508-016-0747-x 27178173 10.1007/s10508-016-0747-x PMC5107348

